# Concomitant Acute Rheumatic Fever and Acute Post Streptococcal Glomerulonephritis

**DOI:** 10.7759/cureus.16357

**Published:** 2021-07-13

**Authors:** Somshukla Ghosh, Kelli King-Morris, Joshua Shultz

**Affiliations:** 1 Internal Medicine, University of Central Florida College of Medicine, Orlando, USA; 2 Nephrology, Orlando Veterans Affairs Medical Center, Orlando, USA

**Keywords:** acute rheumatic fever, acute post streptococcal glomerulonephritis, rheumatic heart disease, antibiotic prophylaxis, group a β-haemolytic streptococci

## Abstract

Acute rheumatic fever (RF) and acute post Streptococcal glomerulonephritis (APSGN) are non-suppurative complications of a Group A Streptococcus (GAS) infection. The concomitant incidence of both complications in a patient is rare because nephritogenic and rheumatogenic strains belong to different serotypes of Group A beta-hemolytic Streptococcus (GABHS). We present a case of a 47-year-old female who had concomitant acute RF and APSGN from a *Streptococcus pyogenes* infection. It is important to have a high clinical suspicion for the sequela of GABHS infection in the setting of cardiac and renal disease following upper respiratory infection (URI) symptoms even in adults and in geographic locations with the nearly undetectable burden of acute RF because of the importance of secondary prophylaxis with an antibiotic.

## Introduction

Acute rheumatic fever (RF) is a non-suppurative complication secondary to tonsillopharyngitis caused by *Streptococcus pyogenes* and acute post Streptococcal glomerulonephritis (APSGN) is a type III hypersensitivity reaction usually following tonsillopharyngitis or skin infection caused by *S. pyogenes* [1]. It is extremely rare for a patient to have concomitant acute RF and APSGN following a Streptococcal tonsillopharyngitis because the rheumatogenic and nephritogenic strains of Group A beta-hemolytic Streptococcus (GABHS) are different. However, such cases have been reported, and can be explained by co-infection with both rheumatogenic and nephritogenic strains.

The United States has a very low incidence of acute RF and it is no longer a reportable disease [2]. Acute RF occurs most commonly in children aged between 5 and 15 years. We present a case of acute RF with concurrent APSGN in an adult with no travel history outside of the United States in more than a decade. 

Timely diagnosis of acute RF is critical because of the need for secondary prophylaxis with an antibiotic to avoid further valvular damage with a subsequent GABHS infection. This is because patients with a subsequent GABHS infection are at a higher risk for a recurrent attack for acute RF and the severity of rheumatic heart disease increases with subsequent episodes. Also, timely diagnosis and early treatment of APSGN are important for a reduction in morbidity.

## Case presentation

A 47-year-old female with no significant past medical history, including RF in childhood, presented to the emergency department (ED) with the chief complaint of acute onset respirophasic chest pain for two days. The pain was described as sharp in quality, 7/10 in intensity, unrelated to rest or activity, and non-radiating. She also complained of generalized fatigue, bilateral shoulder and knee pain, bilateral leg swelling, and dark frothy urine, all of which started a few days prior to the onset of chest pain. Other review of systems was negative. She reported a sore throat which started three weeks prior to admission and lasted approximately one week before spontaneously resolving. The patient was originally from Honduras; however, she had no history of travel outside of the United States in the past 14 years. She was living in Florida during the same period. Of note, her children were sick with pharyngitis a month prior to her presentation, however, they did not seek medical treatment. 

**Figure 1:**
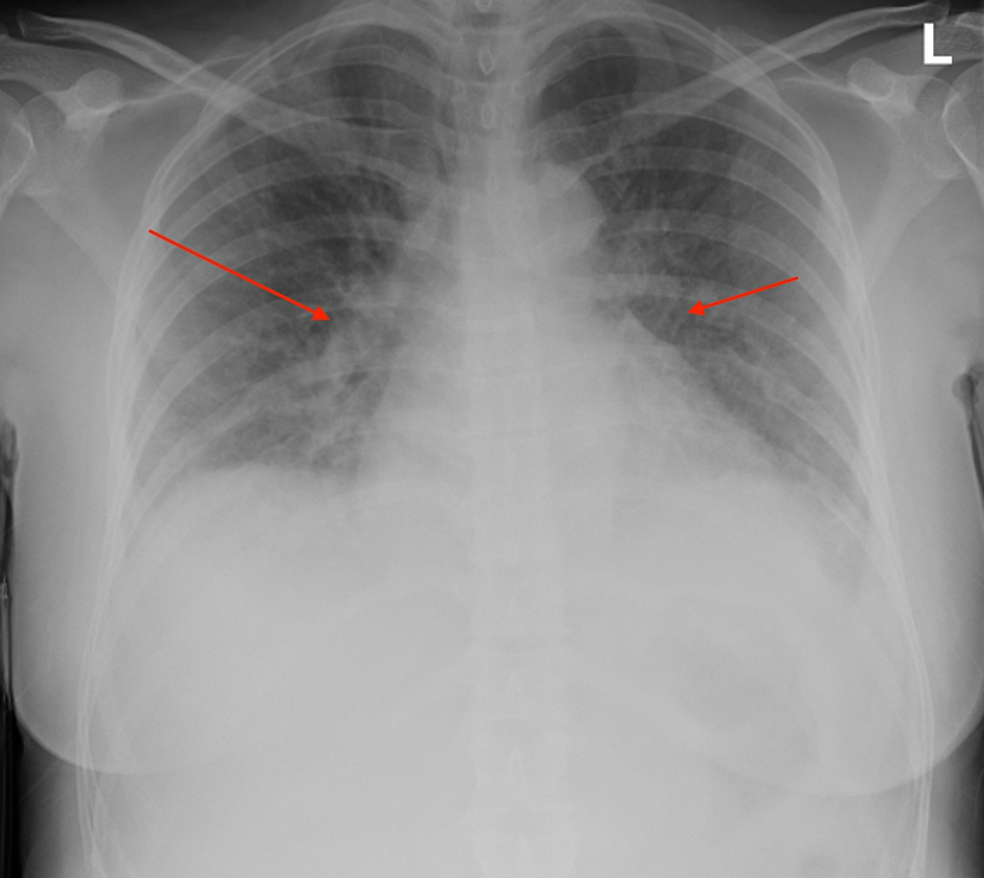
Chest X-ray showing bilateral interstitial edema.

**Figure 2:**
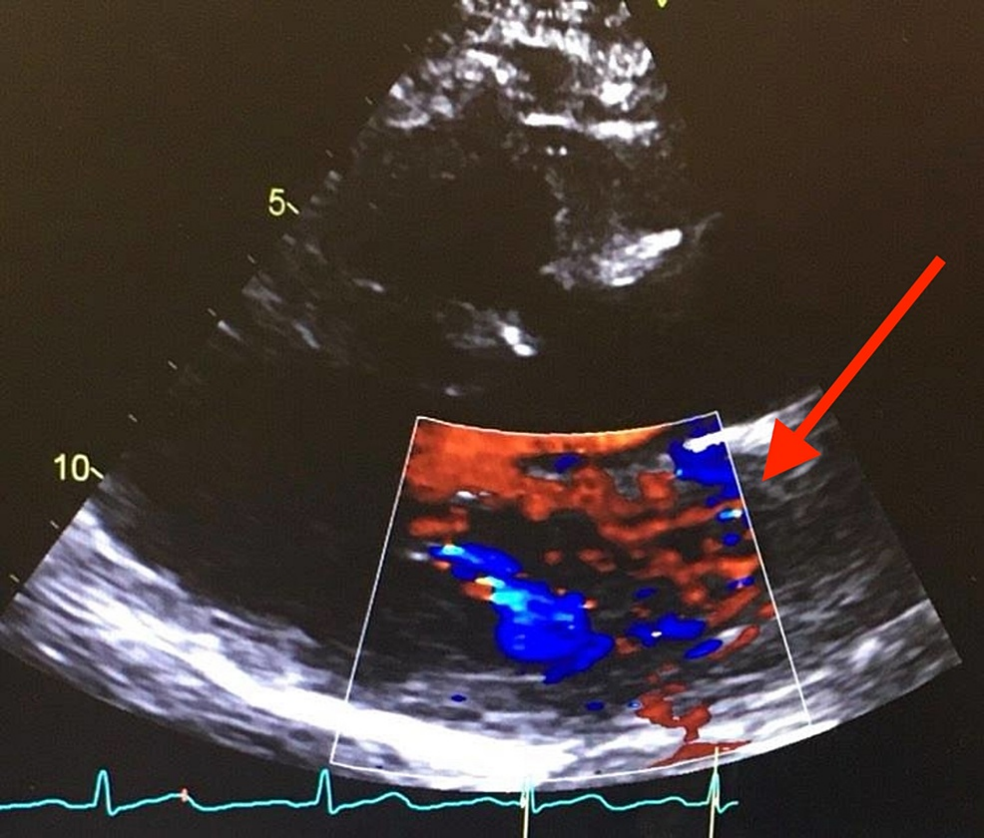
Parasternal long-axis view of echocardiogram showing severe mitral regurgitation.

On physical examination, her temperature was 98.2°F, blood pressure was 160/90 mmHg, heart rate was 84 beats/min, respiratory rate was 12 breaths/min, and oxygen saturation was 99% on ambient air. Cardiopulmonary examination revealed bilateral diffuse lung crackles, normal S1 and S2, a holosystolic 3/6 murmur at the cardiac apex which radiated to her axilla. She also had bilateral 2+ pitting pedal edema. Examination of the skin was normal without any evidence of pyoderma or cutaneous abscess. Labs were significant for creatinine of 1.57 mg/dL, mildly elevated troponin of 0.14 ng/mL, elevated brain natriuretic peptide (BNP) of 16667 pg/mL, and erythrocyte sedimentation rate (ESR) of 69 mm/h (Table 1) . The chest X-ray showed bilateral interstitial edema (Figure 1) and the echocardiogram showed severe mitral regurgitation (Figure 2), grade 1 diastolic dysfunction, and ejection fraction of 55%-60%.

 

Group A Streptococcal antigen was negative, but antistreptolysin O (ASO) antibody was positive at 900 IU/mL (Table 1). 

**Table 1:** Pertinent laboratory investigations. BNP, brain natriuretic peptide; ESR, erythrocyte sedimentation rate; LDL, low-density lipoprotein; ANA, anti-nuclear antibody; ANCA, anti neutrophilic cytoplasmic antibodies; ASO, anti-streptolysin O

Laboratory investigation	Result (reference range)
Serum creatinine	1.57 mg/dL (0.55-1.3)
Troponin	0.14 ng/mL (0-0.03)
BNP	16667 pg/mL (0-450)
ESR	69 mm/h (0-20)
Serum albumin	2.9 g/dL (3.4-5.0)
ASO antibody	>900 IU/mL (<408)
Total cholesterol	174 mg/dL (<200)
LDL	107 mg/dL (<100)
Rheumatoid factor	Negative
ANA	Negative
Cytoplasmic ANCA	<0.2 AI (0.0-0.9)
p-ANCA	<0.2 AI (0.0-0.9)
Double strand DNA antibody	1.0 IU/mL (0-4.9)
Anti-glomerular basement antibody	7 units (0-20)
Complement C3	<40 mg/dL (90-180)
Complement C4	19.30 mg/dL (14-44)
HIV (1 and 2) antigen and antibody screen	Non-reactive
Hepatitis B surface antigen	Negative
Hepatitis C antibody	Negative

Based on her history, physical examination and laboratory, there was a concern for acute RF. We utilized the revised Jones criteria: evidence of Streptococcal infection with positive ASO antibody, one major criterion with carditis, and two minor criteria with elevated ESR and arthralgia. The diagnosis of acute RF was confirmed.

In the setting of elevated creatinine, frothy urine and pitting pedal edema, there was also a concern for a nephrogenic process. The urinalysis was positive for 3+ proteinuria and red blood cells (RBC) of 50-100/hpf, urine protein to creatinine ratio was 8000 mg/g. Serum albumin was 2.9 g/dL, total cholesterol and low-density lipoprotein (LDL) were 174 and 107 mg/dL respectively. She was screened for HIV, Hepatitis B and C, all of which were negative. Rheumatoid factor, Anti-nuclear antibody (ANA), anti neutrophilic cytoplasmic antibodies (ANCA), double-stranded DNA antibody, and anti-glomerular basement membrane were negative. C4 was normal at 19.30 mg/dL, however, C3 was low at <40 mg/dL (Table 1). 

**Figure 3:**
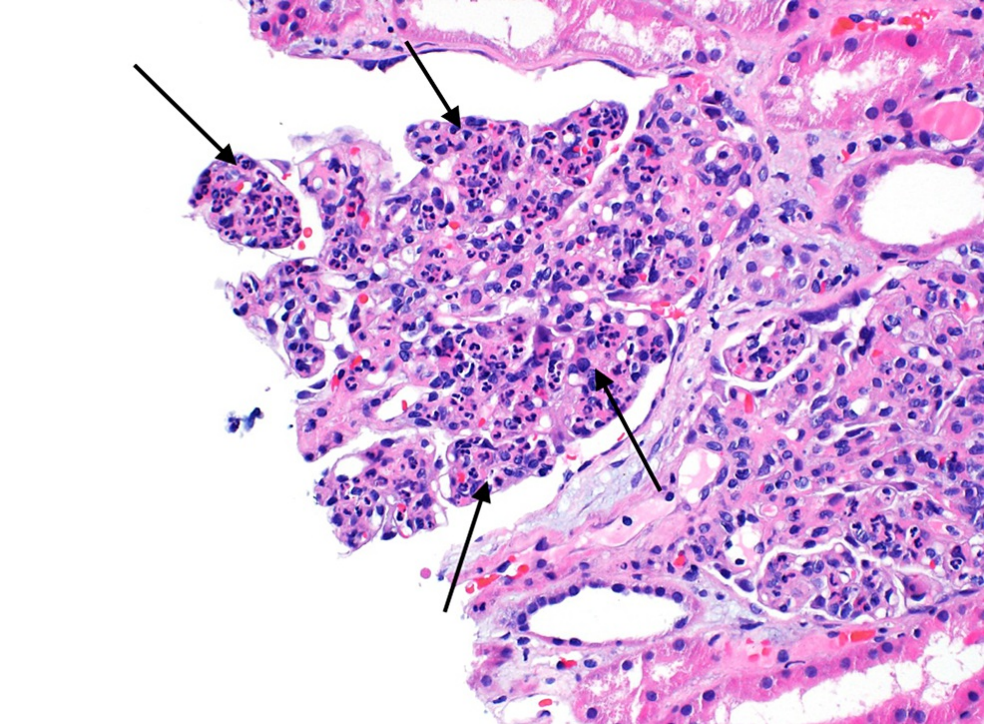
Light microscopy showing diffuse global mesangial and endocapillary hypercellularity composed of prominent neutrophils with lesser amounts of mononuclear cells.

Renal biopsy reported diffuse immune complex mediated proliferative and exudative glomerulonephritis. Specifically, the light microscopy showed enlarged glomeruli with diffuse global mesangial and endocapillary hypercellularity (Figure 3).

**Figure 4:**
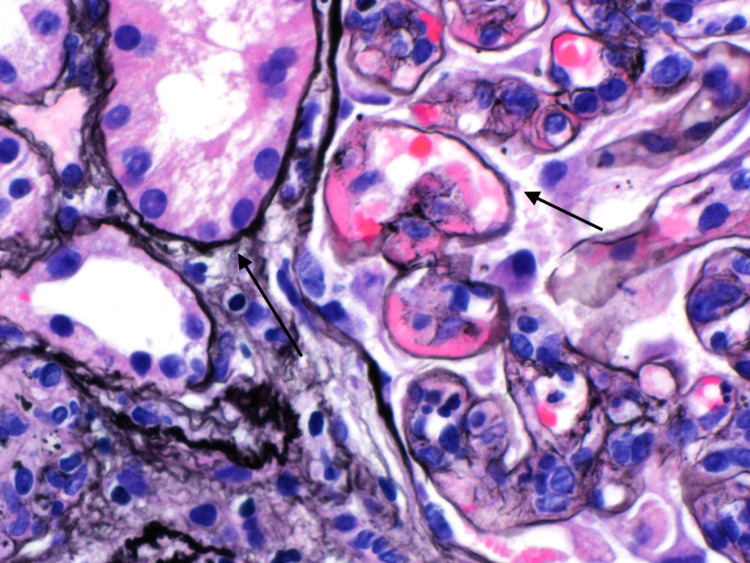
Glomeruli showing segmental subendothelial PAS-positive deposits forming ‘wire loop' like lesions. PAS, periodic acid-Schiff

There were also focal glomeruli showing subsegmental subendothelial periodic acid-Schiff (PAS)-positive deposits forming ‘wire loop’ like lesions (Figure 4). 

**Figure 5:**
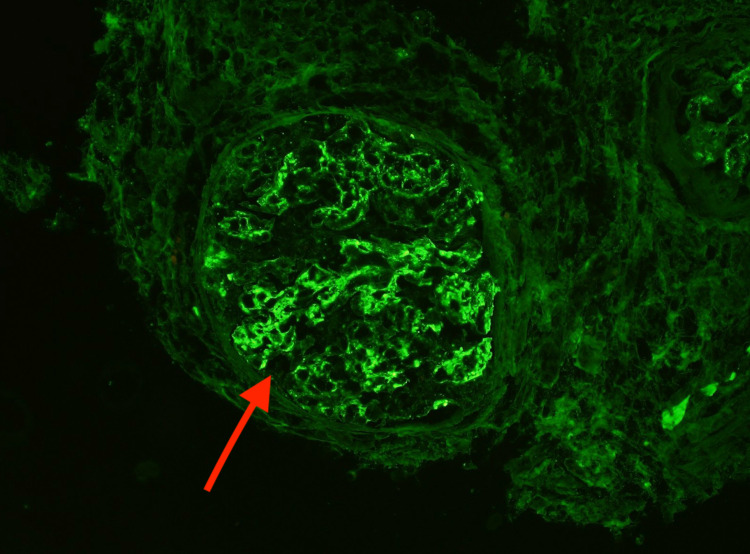
Immunofluorescence microscopy showed glomeruli with global mesangial and capillary loop granular staining by IgG. IgG, immunoglobulin G

**Figure 6:**
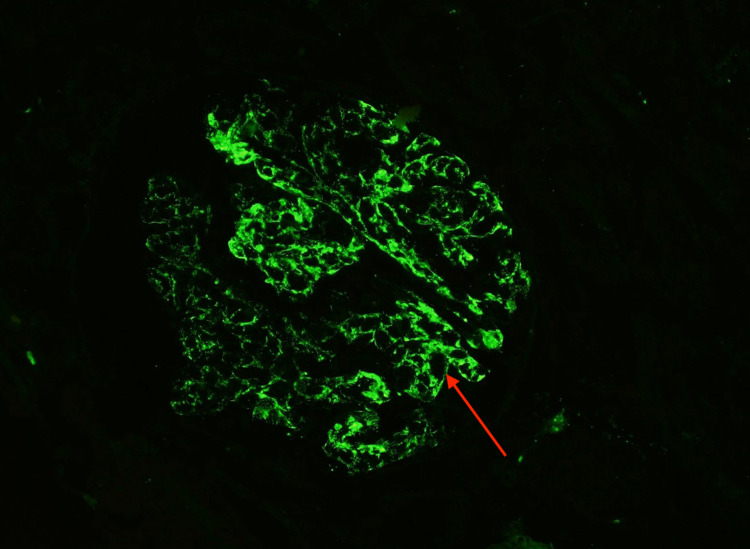
Immunofluorescence microscopy showed glomeruli with global mesangial and capillary loop granular staining by C3.

There was moderate interstitial fibrosis and tubular atrophy, with a mononuclear infiltrate predominately limited to the areas of fibrosis. Immunofluorescence microscopy showed glomeruli with global mesangial and capillary loop granular staining by immunoglobulin G (IgG) (3+) (Figure 5), C3 (3+) (Figure 6), and C1q (1+).

**Figure 7:**
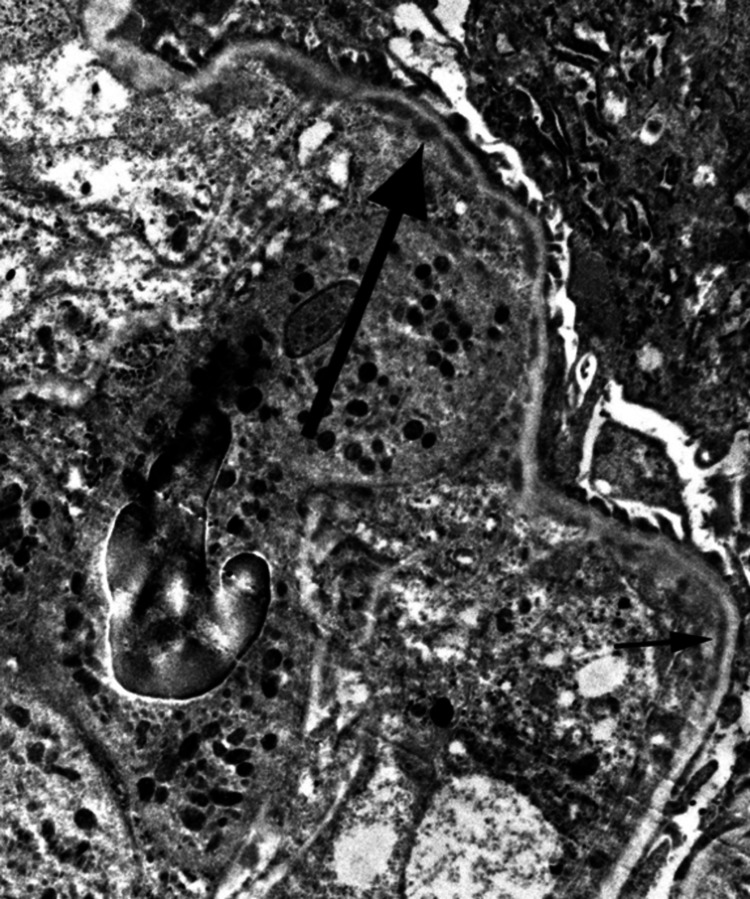
Electron microscopy showing subendothelial deposits.

**Figure 8:**
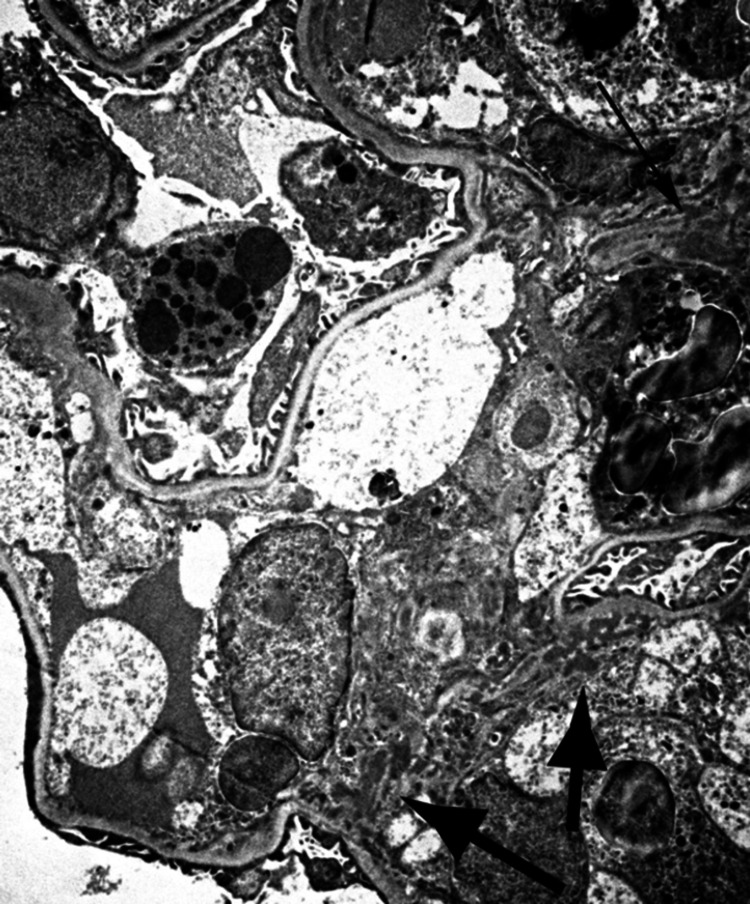
Electron microscopy showing frequent mesangial deposits.

Electron microscopy showed frequent capillary loop hypercellularity. The mesangial region was expanded, hypercellular and showed frequent electron-dense deposits (Figures 7-8).

There was also moderate to severe epithelial foot process effacement.

Given the multiple findings above, the patient was diagnosed to have APSGN concurrently with acute RF. She was started treatment with furosemide and lisinopril. She was also given amoxicillin for the GABHS infection. She was recommended to continue amoxicillin for secondary prophylaxis of GABHS for lifelong. 

## Discussion

Acute RF results from an autoimmune response to Group A Streptococcal pharyngitis. Acute RF is usually a complication secondary to tonsillopharyngitis, caused by *S. pyogenes* M-18, M-1, M-3 serotypes [3]. Acute RF can occur at any age, although most commonly occurs in children aged between 5 and 15 years. Adults presenting with first onset acute RF is extremely rare [4-5]. In our case, the patient was a 47-year-old female presenting with constitutional symptoms and was found to have valvulitis, which is the most common finding among patients with acute RF. 

Acute post Streptococcal glomerulonephritis is a type III hypersensitivity reaction usually following tonsillopharyngitis or skin infection caused by *S. pyogenes*. It is an extremely rare presentation for a patient to have concomitant acute RF and APSGN following a Streptococcal tonsillopharyngitis. However, such cases have been reported, explained by co-infection with both rheumatogenic and nephritogenic strains. Interestingly, our patient had sorethroat preceeding all other symptoms and sore throat tends to be associated with a rheumatogenic strain and a nephrogenic strain is less likely [6].

Another interesting feature to be noted in this patient is that she had nephrotic range proteinuria with renal biopsy findings consistent with APSGN. Only 5% of patients with APSGN are known to have nephrotic range proteinuria [7]. It is unclear if the presence of nephrotic range proteinuria is a feature of concurrent acute RF and APSGN.

In the literature, it is evident that the incidence of such concurrent non-suppurative complication of Group A streptococci (GAS) infection is very rare (possibly less than 30 cases worldwide) and of these most are children and adolescents aged less than 18 years [4]. The most recent reported case of an adult with concurrent acute RF and APSGN is the first reported case from Australia, even in the setting of Australia having a high burden of acute RF [8]. However, historically the United States is a low burden setting for acute RF, and in the last five decades of the 20th century, the incidence of acute RF in the United States has decreased to the extent to which it is no longer a reportable disease. The incidence of acute RF in Florida through 1970-1990 was 5-20/100,000 persons and was one of the pockets of significant incidence of acute RF in the United States. The only states with a higher incidence were California, Tennessee, and Alabama with 20-40/100,000 persons. The incidence of acute RF diminished significantly over the years with only one state (West Virginia) with a measurable incidence at less than 5/100,000 persons [2]. To our knowledge, this is the first reported case of concurrent acute RF and APSGN in Florida. 

Diagnosis of acute RF confers the need for secondary prophylaxis with antibiotics as these patients are at risk for subsequent complications and future GABHS infections which may result in worsening valvular damage. Treatment entails administration of intramuscular Benzathine Penicillin G every four weeks for patients with suspected noncompliance to oral antibiotics. As per the American Heart Association, patients with rheumatic heart disease should receive secondary prophylaxis until 40 years of age or 10 years after their last attack, whichever is longer. However, in patients with continued high risk for GABHS infection, lifelong secondary prophylaxis is warranted. Our patient was recommended to take lifelong secondary prophylaxis with amoxicillin because she already had congestive heart failure from rheumatic heart disease and hence subsequent valvular damage secondary to recurrent acute RF could potentially prove to be lethal. 

Regarding APSGN, delay in onset of treatment is associated with poor outcomes. Timing of diagnosis affects morbidity which is improved with early treatment. Long-term consequences such as hypertension, proteinuria, and hematuria should be monitored for resolution and may take years to resolve [9].

## Conclusions

This case highlights the importance of high clinical suspicion for the sequela of GABHS infection in the setting of cardiac and renal disease that follows upper respiratory infection (URI) symptoms, even in adults and in a geographic location with low incidence of non-suppurative complications of GABHS infection. Timely diagnosis and treatment are critical to prevent morbidity and mortality. Also, secondary prophylaxis against GABHS infection is of prime importance in patients with rheumatic heart disease.
